# Molecular and epidemiological characteristics of human Puumala and Dobrava-Belgrade hantavirus infections, Germany, 2001 to 2017

**DOI:** 10.2807/1560-7917.ES.2019.24.32.1800675

**Published:** 2019-08-08

**Authors:** Mirko Faber, Detlev H Krüger, Brita Auste, Klaus Stark, Jörg Hofmann, Sabrina Weiss

**Affiliations:** 1 Department for Infectious Disease Epidemiology, Robert Koch Institute, Berlin, Germany; 2 Charité-Universitätsmedizin Berlin, corporate member of Freie Universität Berlin, Humboldt-Universität zu Berlin, and Berlin Institute of Health, Institute of Virology, Berlin, Germany; 3 These authors contributed equally and share last authorship

**Keywords:** Hantavirus, Puumala virus, Dobrava-belgrade virus, Molecular epidemiology, HFRS, Disease Notification, Environmental Exposure, Zoonoses, Phylogeography, Outbreak, Humans, Central Europe, Germany

## Abstract

**Introduction:**

Two hantavirus species, Puumala (PUUV) and Dobrava-Belgrade (DOBV) virus (genotype Kurkino), are endemic in Germany. Recent PUUV outbreaks raised questions concerning increasing frequency of outbreaks and expansion of PUUV endemic areas.

**Aims:**

To describe the epidemiology of human PUUV and DOBV infections in Germany.

**Methods:**

We conducted an observational retrospective study analysing national hantavirus surveillance data notified to the national public health institute and hantavirus nucleotide sequences from patients collected at the national consultation laboratory between 2001 and 2017. Matching molecular sequences with surveillance data, we conducted epidemiological, phylogenetic and phylogeographic analyses.

**Results:**

In total, 12,148 cases of symptomatic hantavirus infection were notified 2001–17 (mean annual incidence: 0.87/100,000; range: 0.09–3.51). PUUV infections showed a highly variable space-time disease incidence pattern, causing large outbreaks every 2–3 years with peaks in early summer and up to 3,000 annually reported cases. Sex-specific differences in disease presentation were observed. Of 202 PUUV nucleotide sequences obtained from cases, 189 (93.6%) fall into well-supported phylogenetic clusters corresponding to different endemic areas in Germany. DOBV infections caused few, mostly sporadic cases in autumn and winter in the north and east of Germany.

**Conclusions:**

The frequency of PUUV outbreaks increased between 2001 and 2017 but our data does not support the suggested expansion of endemic areas. The epidemiology of PUUV and DOBV-Kurkino infections differs in several aspects. Moreover, the latter are relatively rare and combining efforts and data of several countries to identify risk factors and develop specific recommendations for prevention could be worthwhile.

## Introduction

Hantaviruses are a diverse group of small mammal-associated viruses with a worldwide distribution. Depending on whether human infections are caused by New- or Old- World hantaviruses, the disease is characterised by different clinical pictures and described as Hantavirus (Cardio-)Pulmonary Syndrome (HPS/HCPS) in the Americas and Haemorrhagic Fever with Renal Syndrome (HFRS) on other continents. Sometimes the term Nephropathia epidemica (NE) is used to describe the epidemic occurrence of renal disease without haemorrhagic symptoms that are linked to infections with strains occurring in the northern and central part of Europe, specifically Puumala virus (PUUV). In clinical practice, syndromes are not always as clear cut as their names suggest and pulmonary and renal complications can also occur in HFRS and HPS/HCPS, respectively [1-3].

Hantavirus infections are typically zoonotic. The viruses are carried by small mammals and are transmitted to humans through inhalation of dust contaminated with saliva, faeces or urine of infected animals or through bites and probably also by ingestion of contaminated food [4]. All known human pathogenic hantaviruses are hosted by specific rodent species, however, novel hantaviruses have also been detected in shrews, moles and bats [5]. Some hantavirus species can cause infection where the case fatality ratio can be up to 50% [1,6-8].

Most cases of hantavirus disease are reported from China, which had an annual mean of 13,809 cases between 2004 and 2015 corresponding to an incidence of 0.98 cases per 100,000 population [9]. In Europe, two known hotspots of infection are Finland with an average of 1,500 cases annually and morbidity as high as 30 per 100,000 population [10,11] and Russia with around 7,500 cases annually on average and morbidity of around six per 100,000 population [10,12]. Since 2001, when hantavirus disease became notifiable in Germany, up to 3,000 reported cases have been observed annually; making Germany an additional hotspot of hantavirus disease in Europe. Besides the dominating Puumala virus (PUUV) associated with the bank vole (*Myodes glareolus*), the Dobrava-Belgrade (DOBV) virus (type Kurkino), associated with the striped field mouse (*Apodemus agrarius*), is the second human pathogenic hantavirus endemic to Germany with a different geographical distribution and fewer cases per year [13].

The aim of this study was to describe the epidemiology, space-time variation and clinical features of PUUV and DOBV infections in Germany using data from 17 years of molecular and epidemiological hantavirus surveillance.

## Methods

### Collection of data and materials

#### Surveillance data

Symptomatic hantavirus infections with laboratory confirmation have been notifiable in Germany since 2001. Serological evidence or detection of viral RNA by reverse transcription-PCR (RT-PCR) is reported to the local public health department by the identifying laboratory. The health department completes and verifies case information according to the national surveillance case definition (box). Based on available information, cases are assigned the likely virus species that caused the infection (e.g. PUUV, DOBV or unknown if a differentiation is not possible based on serological results). Information about date of onset, clinical symptoms, hospitalisation, outcome and probable place of infection is requested from the patient or treating physician. Case data are anonymised and electronically transmitted to the state health department and from there to the Robert Koch Institute, the national public health institute in Germany.

Box **Robert Koch Institute reference case definition for hantavirus infections, Germany, 2001–2017 [47]**

**Clinical picture of acute hantavirus disease**, defined as presence of at least one of the following symptoms:

• Fever (>= 38.5 °C)

• Haemorrhagic course of disease

• Renal impairment

• Presence of at least two of the following symptoms:

- Cough

- Diarrhoea

- Dyspnoea

- Headache

- Heart or circulatory failure

- Lung infiltrates

- Muscle-, limb- or backache

- Myopia, acute onset

- Nausea

- Vomiting

**AND laboratory confirmation**, defined as a positive outcome of at least one of the following:

• Presence of hantavirus specific IgM or IgA confirmed by IgG

• Significant rise in hantavirus specific IgG in two consecutive samples

• Detection of hantavirus RNA using NAT

NAT: nucleic acid test.

#### Molecular data

Serum samples of patients diagnosed with hantavirus infections were referred to the national consultation laboratory for hantaviruses (Institute of Virology, Charité Berlin, Germany) by hospitals and physicians through a nationwide alert network. For this report, a total of 214 hantavirus nucleotide (nt) sequences (202 PUUV and 12 DOBV) obtained from samples received between 2001–17 were used for molecular analysis. All sequences have been deposited to GenBank, accession numbers MK770453–MK770458 and MK770459–MK770600.

### Data analysis

#### Surveillance data

This report describes laboratory-confirmed hantavirus infections with clinical symptoms reported 2001–17 as at 1 June 2018. For the data analysis, we applied the national surveillance case definition (box).

Data analysis was conducted using STATA 15 (StataCorp, College Station, Texas, United States) and Microsoft Excel 2010. Statistical tests (chi-squared test or Fisher’s exact test) were used as appropriate. Maps were created using RegioGraph Analyse 16.0 (GfK SE, Nürnberg, Germany).

#### Molecular data

Samples were tested using a molecular screening assay based on the large (L) segment of the hantavirus genome [14] and subsequently Sanger sequenced. PUUV RNA positive samples were further analysed using an assay based on the small (S) segment [15]. Novel sequences were processed in the software package Geneious v10.2 (Biomatters Ltd, Auckland, New Zealand) and compared with those from previous studies [15-17]. Alignments were created by MUSCLE and improved using the gblocks algorithm as implemented in SeaView v4 [18,19]. Maximum likelihood (ML) phylogenetic trees were inferred using the PhyML-SMS server [20] based on alignments of 506 nt and 347 nt for PUUV S and DOBV L segments, respectively. The best-fitting model of nt substitution was identified as HKY85 + G + I for the PUUV S based tree and the TN93 + I model for the DOBV L based tree. Trees were visualised using Fig Tree v1.4 (University of Edinburgh, Edinburgh, United Kingdom). Patristic distances (PD) were calculated using the package ape v4.1 for R [21]. Maps were created using RegioGraph Analyse 16.0 (GfK SE). A phylogenetic cluster is defined as four or more sequences with a maximum patristic distance (PD, evolutionary distances between sequences in a given tree) of 0.21 nt substitutions per site (nss).

## Results

In total, 12,148 cases of hantavirus disease were reported between 2001 and 2017, corresponding to a mean annual incidence of 0.87 cases per 100,000 population (range: 0.09–3.51). Of 12,148 cases, 9,972 (82.09%) had information on the likely virus species causing the infection; 9,714 (97.41%) were notified as PUUV infections, while 223 (2.24%) were notified as DOBV infection (Table 1).

**Table 1:** Notified cases of hantavirus disease by year of notification and likely virus species, Germany, 2001–2017 (n = 12,148)

Virus species	Year																	
	2001	2002	2003	2004	2005	2006	2007	2008	2009	2010	2011	2012	2013	2014	2015	2016	2017	Total
**Notified cases by pathogen**																		
Puumala virus	110	172	106	212	387	57	1,625	204	143	1,873	233	2,370	97	336	519	136	1,134	9,714
Dobrava-Belgrade virus	0	1	0	8	7	2	7	16	10	8	20	33	12	23	27	18	31	223
Hantaan virus	9	6	7	0	1	0	0	0	0	1	0	0	0	2	1	0	4	31
Seoul virus	0	0	0	0	0	0	0	0	0	0	0	0	0	0	0	0	1	1
Sin Nombre Virus	0	0	0	0	0	0	0	0	0	0	0	0	0	0	1	0	2	3
Not specified	63	49	31	22	52	13	55	23	28	134	52	422	52	213	281	127	559	2,176
**Total**	182	228	144	242	447	72	1,687	243	181	2,016	305	2,825	161	574	829	281	1,731	12,148
Incidence^a^	0.22	0.28	0.17	0.29	0.54	0.09	2.05	0.30	0.22	2.47	0.37	3.51	0.20	0.71	1.01	0.34	2.10	0.87
Hospitalised n/N	121/174	121/228	104/144	177/240	318/447	52/72	966/1,685	171/241	134/181	1,369/2,011	239/304	1,980/2,818	110/161	358/451	456/562	202/264	1,127/1,572	8,005/11,555
Hospitalised (%)	69.5	53.1	72.2	73.8	71.1	72.2	57.3	71.0	74.0	68.1	78.6	70.3	68.3	79.4	81.1	76.5	71.7	69.3

^a^ Incidence per 100,000 population.

Other reported virus species included one case with a travel history to Indonesia with a Seoul virus infection confirmed by PCR and sequencing [22] and three Hantaan virus infections after returning from countries in south-east Asia. The additional 31 cases notified as Sin Nombre and Hantaan infections without a corresponding travel history are regarded as erroneous data entries and likely represent additional PUUV or DOBV infections. A total of 2,176 cases were notified not specifying the virus species; the distribution of these cases by time, place and person does not differ from those notified as PUUV or DOBV.

**Figure 1:**
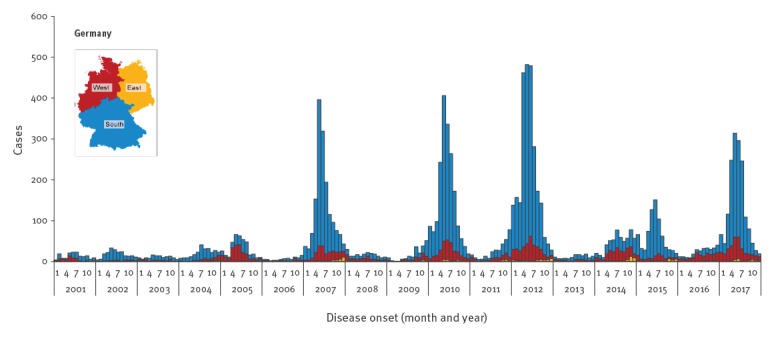
Notified cases of hantavirus disease by month of disease onset and region, Germany, 2001–2017 (n = 11,330)

Between 2001 and 2017, there was geographical and temporal variation in incidence. The years with only few cases alternated with outbreak years showing a steep increase in weekly cases in spring/early summer and a high total case load. With a mean annual incidence of 2.53 per 100,000 population, the four largest outbreak years 2007, 2010, 2012 and 2017, respectively, contributed 8,259/12,148 (68.0%) of the total cases reported between 2001 and 2017. In the remaining 13 years, the mean annual incidence was lower at 0.36 per 100,000 population. Between 2001 and 2017, most cases were notified in the southern part of Germany with fewer cases in the west and only sporadic cases in the east (Figure 1). In total, 8,005/11,555 (69.28%) cases were hospitalised. The annual proportion of hospitalised cases (also an indicator of the overall sensitivity of the surveillance system) showed no clear long-term trend (Table 1).

Inlay shows the location of the regions within an outline of Germany.

**Figure 2:**
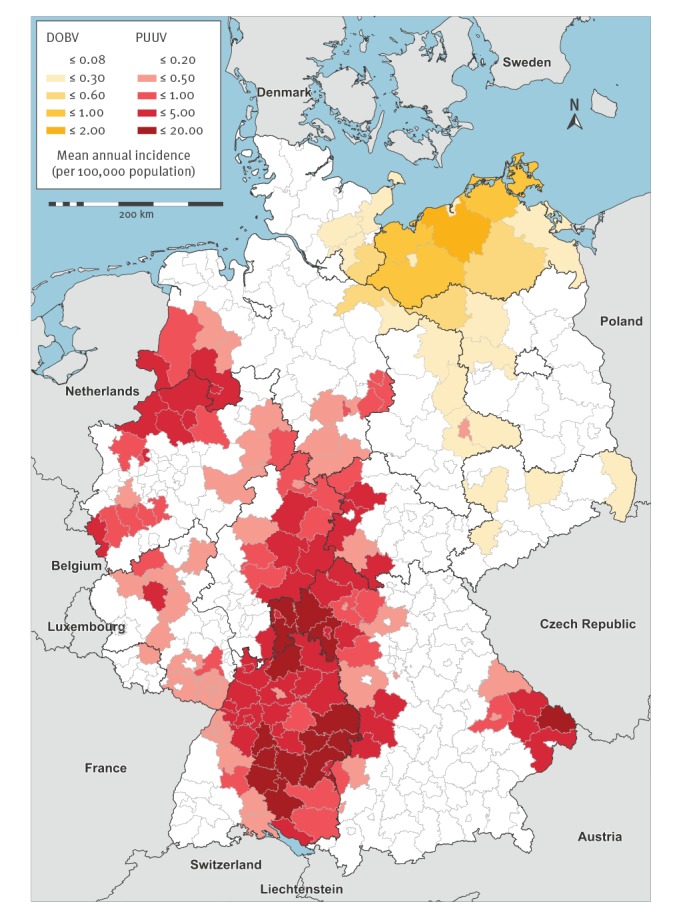
Mean annual incidence of notified cases of hantavirus disease by county of infection and notified virus species, Germany, 2001–2017

PUUV: Puumala virus; DOBV: Dobrava-Belgrade virus.

Of 11,261 cases with information on the likely place of infection, 10,974 (97.45%) had likely acquired the infection in Germany while 228 (2.02%) and 59 (0.52%) infections were likely acquired in other European and non-European countries, respectively. Within Germany, hantavirus infections predominantly occurred in well-known PUUV endemic areas e.g. the Swabian Jura in the state of Baden-Württemberg, the Bavarian Forest in the state of Bavaria or the Münster/Osnabrück region at the border of North-Rhine-Westphalia and Lower Saxony. DOBV infections were notified predominantly from counties in the north and the east of Germany. Figure 2 maps the mean annual incidence over 17 years. During outbreak years, however, several counties in PUUV endemic areas reached annual incidences between 50 and 90 per 100,000 population.

**Figure 3:**
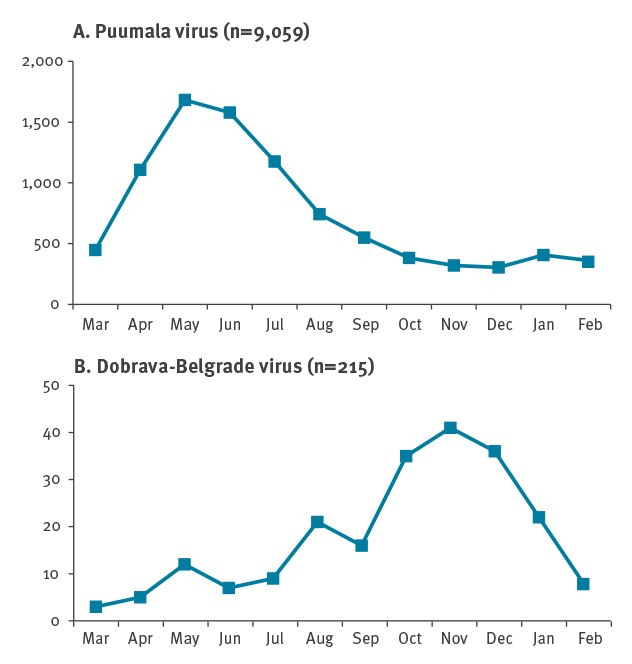
Notified cases of hantavirus disease by notified virus species and month of disease onset, Germany, 2001–2017 (n = 9,274)

Cases of PUUV infections (by month of disease onset) typically started to increase in late winter/early spring, peaked in May/June before decreasing through late summer and autumn (Figure 3A). Rising case numbers in autumn and winter months were typically followed by large PUUV outbreak years. In contrast, DOBV infections occurred mostly during autumn and winter (158/215; 73.49%) with a peak in November (Figure 3B).

**Figure 4:**
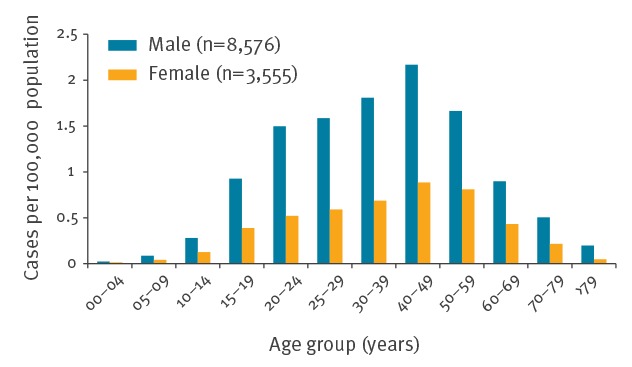
Mean annual incidence of notified cases of hantavirus disease by age group and sex, Germany, 2001–2017 (n = 12,131)

Age-specific incidence was highest in persons aged 20–60 years old, peaking in the age group 40–49 (mean annual incidence 1.54/100,000 population). In all age groups, incidence among men was higher compared with women, 2.51-fold over all age groups (Figure 4).

Fever, renal impairment, headache and muscle-, limb- or backaches were the most often reported symptoms among notified cases (all present in > 50% of cases). Renal impairment was more frequently reported among male compared with female patients, while headache, nausea, vomiting and acute onset of myopia was more frequent among female patients (all p < 0.01, respectively Table 2).

**Table 2:** Absolute and relative frequency of symptoms by sex, notified hantavirus cases, Germany, 2001–2017 (n = 12,131)

Symptom	Male		Female		P value^a^
	n	%	n	%	
Fever (>= 38.5 °C)	7,423	86.6	3,064	86.2	NS
Renal impairment	5,827	68.0	2,295	64.6	< 0.01
Muscle-, limb- or backache	5,583	65.1	2,329	65.5	NS
Headache	4,536	52.9	2,069	58.2	< 0.01
Nausea	2,036	23.7	1,120	31.5	< 0.01
Vomiting	1,553	18.1	846	23.8	< 0.01
Diarrhoea	1,159	13.5	467	13.1	NS
Cough	934	10.9	411	11. 6	NS
Dyspnoea	437	5.1	217	6.1	NS
Myopia, acute onset	415	4.8	221	6.2	< 0.01
Heart or circulatory failure	150	1.8	70	2.0	NS
Lung infiltrates	66	0.8	37	1.0	NS
Haemorrhagic course of disease	17	0.2	11	0.3	NS
Total	8,576	100	3,555	100	NA

NA: not applicable; NS: not statistically significant.

^a^ Fisher’s exact p value.

In six cases, an acute hantavirus infection may have contributed to the death of the patient. These included two haemorrhagic courses of PUUV infection in patients aged 65 years and older and three non-haemorrhagic courses of PUUV infection in patients with co-morbidities. An additional death occurred in a young man with DOBV (genotype Sochi) infection (confirmed by sequencing) after travel to the Caucasus region.

### Phylogenetic and phylogeographic analyses

**Figure 5:**
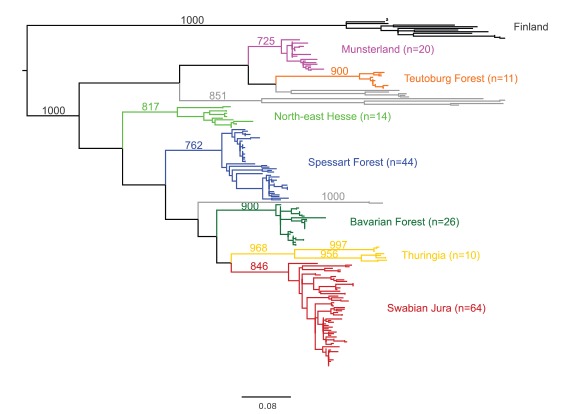
Phylogenetic analysis of Puumala virus sequences in samples referred to the German consultation laboratory, Germany, 2004–2017

^a^ Marks a sequence obtained from a patient with a travel history to Finland (07/H317).

Viral nt sequences were obtained from samples referred to the national consultation laboratory for hantaviruses between 2004 and 2017. Sequences from 202 patients were used for molecular analysis of PUUV, 59 of these from the 2017 epidemic. Of these, 189/202 (93.6%) fall into well-supported phylogenetic clusters (Munsterland, Teutoburg Forest, North-east Hesse, Spessart Forest, Bavarian Forest, Thuringia, Swabian Jura; Figure 5). The average PD within one cluster in the present dataset is 0.05 nss. Sequences within one of these geographical clusters show a pairwise identity of > 90%, whereas sequences from different clusters range from 81% (between Munsterland and Spessart Forest) and 87.9% (between Munsterland and Bavarian Forest).

Colour indicates putative geographic clusters as defined in the text; grey branches refer to sequences not yet assigned to any clear-defined phylogeographic cluster (i.e. < four sequences per region).

Maximum likelihood (ML) tree based on a 506 nt alignment of partial S segments of Puumala virus. ML tree was calculated using the HKY85 + G + I model of nt substitution; scale bar indicates nt substitutions per site. Bootstrap values are given for relevant clades, if above 750 (75%).

**Figure 6:**
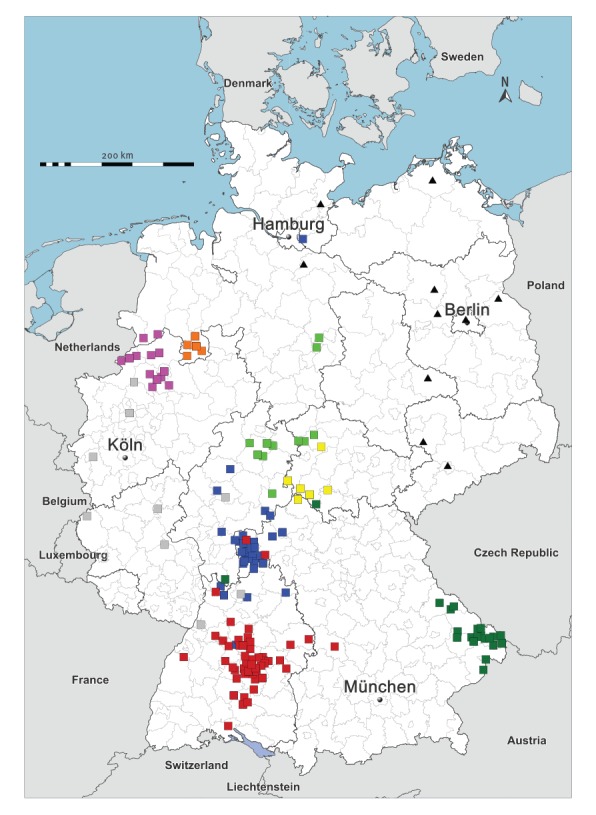
Place of residence of cases of hantavirus disease with available hantavirus nt sequences, Germany, 2004–2017 (n = 210)

Figure 6 shows the place of residence of all patients with PUUV infections between 2004 and 2017 where information was available. PUUV cases are indicated as squares coloured according to phylogenetic clusters in Figure 5. Of 189 cases, the sequence in 180 (95.24%) cases belonged to a cluster of sequences from patients of the same area of residence. These include three cases where the sequence clustered with sequences from an area the patient had travelled to before symptom onset. For one sample (12/H463), no information on residency was available. In nine cases geographical and phylogenetic clusters were discrepant, but the putative place of infection was mostly within short geographic distance.

Cases caused by Puumala virus are depicted by squares coloured according to phylogenetic clusters (Figure 5). Grey squares refer to sequences not yet assigned to any clear-defined phylogeographic cluster (i.e. < 4 sequences per region). Dobrava-Belgrade virus–infected cases are depicted as black triangles.

**Figure 7:**
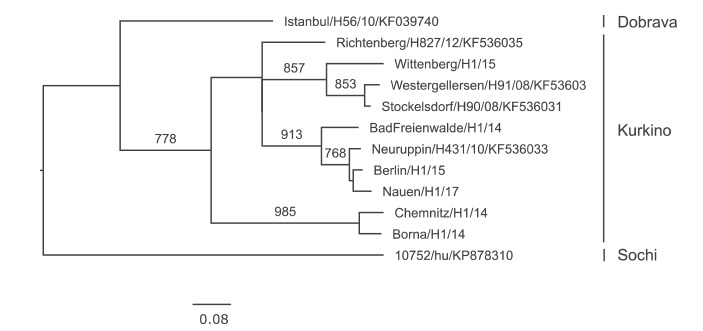
Phylogenetic analysis of Dobrava-Belgrade virus sequences in samples referred to the German consultation laboratory, Germany, 2004–2017

Since 2008, DOBV sequences could be amplified from 12 human samples referred to the national consultation laboratory (Figure 7). One sequence was obtained from a patient hospitalised in Istanbul, Turkey and was identified as DOBV genotype Dobrava [23]. Another sequence was amplified from a Russian patient hospitalised in Heidelberg and was clearly distinct from all other European DOBVs, belonging to the DOBV genotype Sochi [24]. The remaining 10 sequences were identified as DOBV genotype Kurkino. All patients carrying the Kurkino genotype were living in the north-eastern part of Germany.

Maxiumum-likelihood (ML) tree based on a 347 nt aligment of partial L sequences of Dobrava-Belgrade virus. Scale bar gives nt substitutions per site; bootstrap values above 750 (75%) are given above branches. ML tree was calculated using the TN93 + I model.

## Discussion

We analysed data from 17 years of epidemiological and molecular hantavirus surveillance in Germany. We found pronounced space-time variability in disease incidence, clear but divergent seasonality of symptomatic PUUV and DOBV infections, distinct geographical clusters of PUUV strains and differences in disease presentation between women and men.

The complexity of hantavirus epidemiology is highlighted by the spatio-temporal variation of disease in the population. It is driven by interactions between the virus, reservoir animals, humans as a dead-end host and factors such as weather and climate influencing environmental conditions and human and animal behaviour [25,26]. In Germany, the principal geographical distribution of PUUV- and DOBV endemic areas, as indicated by both serology-based surveillance data and our phylogeographic analysis of hantavirus nt sequences, can be explained by the distribution of the respective reservoir animals (the western evolutionary lineage of the bank vole and the striped field mouse, respectively) [27]. While no statistically significant differences were observed in terms of the age- and sex distribution of the affected population and the course of disease, hantavirus epidemiology in PUUV and DOBV endemic areas differ fundamentally in other aspects: The typical ‘outbreak years’ and vastly fluctuating annual case numbers are only apparent in the regions where PUUV infections occur. In contrast, the number of annually reported cases due to DOBV infection is comparably stable (12–31 cases annually between 2013 and 2017). The climatic and ecological factors leading to marked oscillations in the population density of *M. glareolus* seem to be much less important for members of the *Apodemus* genus, the DOBV reservoir. Moreover, the main seasonal periods for virus transmission to humans (disease incidence peaks in May/June and October–December for PUUV and DOBV infections, respectively) differ. This suggests that the period of highest population density and infection prevalence in the respective reservoir animal and/or the contact pattern with humans is different for PUUV and DOBV infections. Risk-factors and prevention measures established for PUUV infections [28,29] might, therefore, not be targeting DOBV infections.

The epidemiology of PUUV in endemic areas and underlying factors have been studied extensively and much of the variation in disease incidence in central and western Europe may be explained by population increases of the bank vole following masting events of the common beech (*Fagus sylvaticus*) and other seed-producing trees/plants [30]. Some authors have proposed statistical models to predict outbreak years in PUUV endemic areas. Most of these models take into account the regional intensity of beech mast [31,32], which is a major factor driving overwintering efficiency and population density of the bank vole. Others consider measurements of rodent density of or even virus prevalence in the reservoir, which requires labour-intensive field- and laboratory work [33] but allows assessment of more direct influences of the probability of virus transmission to humans. More recently, models relying on climate/weather and habitat (e.g. density of deciduous forests) data alone, have been proposed for outbreak prediction [34]. These predictions can be helpful in raising awareness and preparedness for the disease and a coming increased risk of infection. However, as our data show, predicting whether the following year will be an outbreak year seems also possible by merely observing an increase in weekly case numbers through autumn and winter.

The distribution of our cases by age and sex show a higher incidence among males compared with females and a peak incidence among those aged 30–59 years, confirming prior findings from Germany and other European countries [35,36]. Apart from sex-specific differences in the exposure to pathogens, women and men also differ in their physiological responses to infections [37]. This is in line with our finding of statistically significant differences in the frequency of clinical symptoms in females and males, e.g. renal impairment (more frequent among male cases), headache, nausea, vomiting and acute onset of myopia (more frequent among female cases). In a study overseeing 108 well-characterised patients from Germany, Krautkrämer and colleagues [38] did not observe major differences in disease severity between males and females, while studies from Sweden observed a higher case fatality rate among women [39].

While changing climate conditions might have increased the frequency of strong masting events leading to large outbreaks as observed during the past years [40], PUUV endemic areas in Germany seem stable. Comparison of all available human PUUV sequences from German patients between 2004 and 2017 shows that the vast majority falls into distinct clusters that can be assigned to specific geographical areas and that those clusters and areas remained stable during the past 13 years. The existence and stability of these geographic clusters (including clusters of neighbouring areas) indicate long-term separate evolution of strains in the animal reservoir and argue against a rapid expansion of areas where the virus is present in the environment. This is in line with our previous investigation of an outbreak of PUUV in the state of western Thuringia where the phylogenetic analysis suggested the steep rise in human cases was most likely caused by a proliferation of the population of infected voles rather than a recent spread of the virus from other areas [41].

Given the strong geographic association within > 90% of our hantavirus sequences and the close phylogenetic relationship between human and rodent derived sequences from the same area [15-17,42], there are strong indications that human infections commonly occur close to the cases homes. The few discrepancies observed between phylogenetic cluster and the respective geographic location can be explained by the daily commute or travel history as reported by the case or are compatible with short (day) leisure trips. Using phylogenetic inference on our dataset, we were also able to identify sequences that do not originate in Germany, as shown for sample 07/H317. The patient had been exposed to rodents in Finland before falling ill, which is consistent with sequence data of the virus that was clearly distinct from German sequences [43]. Nine sequences that do not belong to any of the well-defined geographic clusters segregate into four distinct and well supported phylogenetic clades (> 98% bootstrap support). These might be affirmed as new geographic clusters once additional sequences from the corresponding regions become available. For those strains, rodent sequences from the same area would be desirable for confirmation. In order to investigate the possibility of reassortment events, which are known to occur in the rodent reservoirs [44], full-genome sequences of patients infected with PUUV should be obtained. However, this is challenging due to the typically very low viral RNA concentrations in the patient’s blood and short viraemia.

In this study, we used serological as well as sequencing-based data. Due to the large serological cross-reactivity between virus species, typing based on serology alone can be misleading in an individual patient [45]. The overall concordance of PUUV and DOBV endemic areas determined through routine surveillance and sequencing data, however, was high and suggests that serology-based typing can be useful for surveillance purposes. Still, continuous sequencing and phylogenetic analysis of circulating strains is necessary to detect the emergence of new strains (or the lack thereof) and is recommended to be done in at least a subsample of patients.

### Conclusions

The presented data from 17 years of hantavirus surveillance and research provides important insights into the epidemiology of both hantavirus species endemic in Germany. While in the past 10 years several exceptionally large PUUV outbreaks have occurred in Germany, and the overall frequency of these outbreak years occurring might have increased due to climate factors, our data argues against an expansion of areas where the virus is present in the environment. Transmission routes and risk factors for human PUUV infections have been thoroughly investigated in several countries and studies but such studies are urgently needed for DOBV genotype Kurkino infections in order to develop DOBV-specific recommendations for prevention. In contrast to infections with the DOBV-Dobrava genotype in south-east Europe, which are well studied, these infections are relatively rare and it may be worthwhile to pool data of several European countries, including Russia where this virus has been found in rodents and humans [46]. Regarding the clinical picture of hantavirus infections, we recommend medical practitioners to take into account that infections in men and women might present differently. Our data suggests that hantavirus infections are often acquired close to the home of the patient. In order to identify locally increased risks of infection and to better target public health recommendations, it would be helpful to collect and analyse case data of higher geographical resolution than just on a county level.
